# The traditional experience strategy (TES) and combined ultrasonography examination (CUE) for the treatment of lateral compression type 1 pelvic fractures: a historical control study

**DOI:** 10.1186/s12891-021-03993-4

**Published:** 2021-01-25

**Authors:** Hai Huang, Bin-Fei Zhang, Ping Liu, Hong-Li Deng, Peng-Fei Wang, Hu Wang, Bao-Feng Li, Yu-Xuan Cong, Yan Zhuang

**Affiliations:** Department of Orthopedic Trauma, Honghui Hospital, Xi’an Jiaotong University, Beilin District, No. 555 Youyi East Road, Shaanxi Province 710054 Xi’an, Republic of China

**Keywords:** Ultrasonography, Stability, LC-1 pelvic fractures

## Abstract

**Background:**

It is difficult to judge the stability of lateral compression type-1 (LC-1) pelvic fracture, as it is often based on static images of the pelvis. Compared with the traditional experience strategy, ultrasonography examination may be able to distinguish operative and conservative patients before definitive treatment. However, in previous studies, we have not compared the outcomes between traditional experience strategy (TES group) and combined ultrasonography examination (CUE group). Thus, the aim of the study is comparing the differences between TES and CUE strategy, to identify the value of ultrasonography examination.

**Methods:**

Medical records system for patients with LC-1 pelvic fractures who were treated with TES and CUE strategy were included. Patients’ baseline characteristics, treatment strategy, and function were recorded at follow-up. Functional outcomes were evaluated using the Majeed grading system.

**Results:**

In total, 77 patients with LC-1 pelvic fractures were included in the study. There were 42 and 35 patients in TES and CUE group, respectively. Compared to TES group (69 %), there were less proportion patients chosen the operative treatment in CUE group (43 %, *P* = 0.021). The volume of intraoperative blood loss in CUE operative group was more than TES operative group (*P* = 0.037). There were more patients with complete sacral fracture in CUE operative group than TES operative group (*P* = 0.002). The Majeed scores in CUE conservative group was higher than TES conservative group (*P* = 0.008). The overall Majeed scores in CUE group was higher than that in TES group (*P* = 0.039).

**Conclusions:**

The ultrasonography examination could relatively accurately identify the unstable LC-1 pelvis than the traditional experience strategy, the operative rate could be reduced and the overall function of LC-1 patients could be improved under the ultrasonography examination.

**Level of evidence:**

Level III.

## Background

Lateral compression type-1 (LC-1) pelvic fractures are the most common type of pelvic fractures, accounting for approximately 50 % of all pelvic ring fractures [1]. Traditionally, LC-1 pelvic fractures have been defined as rotationally unstable and vertically stable. Most of these fractures could be conservatively treated to achieve a good functional outcome [2–4].

Even though computed tomography (CT) could provide the three-dimensional image and assist treatment [5], it is difficult to judge the stability of every LC-1 pelvic fracture, as it is often based on static images of the pelvis and the treatment strategy was often relied on these images. In fact, Ma et al. found pelvic fracture displacement tended to be underestimated. The peak compression can be 1.3–2.2 times of final compression appearing on images in hospital [6], and Beckmann et al. queried 111 OTA members for treatment recommendations on 27 different LC-1 fractures and found very inhomogeneous responses [7].

In the field of stability of LC-1 fracture, Sagi et al. have conducted perfect exploration to confirm the stability of the pelvic ring under general anesthesia [8]. But it is needed to perform anesthesia for everyone under this method, including the stable pelvis (patients who do not need surgery). Because LC-1 pelvic fracture patients needing operative treatment account for a small number of patients, general anesthesia is not the optimal method for every patient. Our team have conducted a preliminary study using ultrasonography examination [9, 10]. After judging the stability, we carried out targeted treatment. Surgical treatment was used for unstable patients, and conservative treatment was given to stable patients. Not only does it not require general anesthesia, but also no radiation, a clear plan can be given before surgery or conservative treatment. This way could avoid so much disadvantages.

Compared with the traditional experience strategy (TES), combined ultrasonography examination (CUE) may be able to distinguish operative and conservative patients before definitive treatment. However, in previous studies, we have not compared the outcomes between TES and CUE strategy. Thus, we propose a hypothesis: CUE strategy could identify the real unstable and stable pelvis, and the overall function of LC-1 patients who used CUE strategy is better than TES strategy. Therefore, we designed this historical control study to identify the value of ultrasonography examination.

## Methods

### Patient screening and selection

Inclusion criteria for the study required that patients met the diagnostic criteria for LC-1 pelvic fractures [11]. The inclusion criteria were (1) age ≥ 18; (2) history of falling from a height, slipping, or a traffic accident; (3) pelvic pain, tenderness, hip dysfunction, and local swelling; (4) diagnosis and fracture type were confirmed using X-rays and CT. X-ray and CT images revealed partially stable fractures with a lateral-compression type 1 injury in the pelvis; (5) operation was underwent by one team; (6) patients in TES group should be discussed the treatment strategy by more than 5 senior orthopaedic surgeons, patients in CUE group should be available the result of ultrasonography on pelvic stability and discussed the treatment strategy by same senior orthopaedic surgeons in our department; and (7) at least 10 months of follow-up.

The exclusion criteria were (1) age < 18; (2) fractures other than LC-1 pelvic fractures; (3) serious soft tissue injury (Morel-Lavallée); (4) serious multiple injuries.

We searched the medical system records for patients with LC-1 pelvic fractures who were treated with TES and CUE strategy. The patients’ record search periods were from August 2015 to January 2017 for the TES group and from October 2016 to December 2018 for the CUE group.

### Treatment strategy

From August 2015 to January 2017, when we identified a LC-1 pelvic fracture, electrocardiographic monitoring was performed, and haemodynamic stability was assessed. If needed, fluid and blood transfusions were administered immediately. For patients in a stable condition, five senior surgeons in our department decided the treatment strategies (operative and conservative treatment) for every patient, based on the mechanism of injury, fracture classification, pain, displacement on X-ray or CT images, and patients’ demand.

**Fig. 1:**
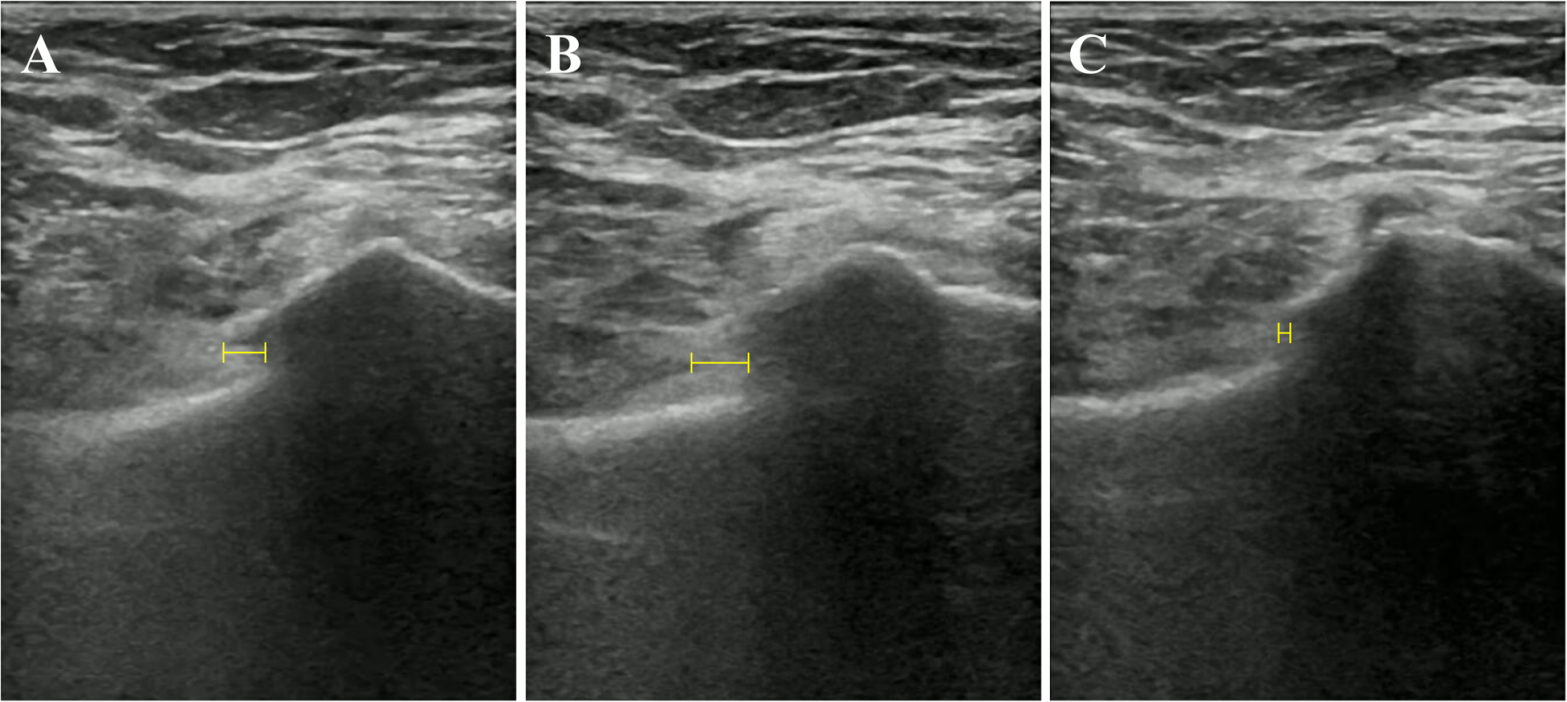
The method used to measure displacement in left-right direction. The yellow line shows the left-right distance. **a** Displacement at rest. **b** Displacement during the compression test. **c** Displacement during the separation test.

From October 2016 to December 2018, when the LC-1 pelvic fracture was identified. After ensuring that the patient’s hemodynamics were stable, pelvic stability was tested using the pelvic compression and separation test on the injured superior pubic ramus. The method and protocol were performed according to our previously published articles [9, 10]. Patients were tested by a senior ultrasound sinologist and orthopedist. Video material was collected from the ultrasound system in order to compare the relative positions of the fracture sites in patients during rest, compression, and separation to determine fracture stability. The detailed formula for calculating mobility had described in the methods section of our previous publications [9, 10]. Figure 1 illustrates the method used to measure displacement in left-right direction. We divided the patients into two groups, a stable group and an unstable group, using left-right mobility ≥ 0.3 cm as the definition of instability [9, 10].

When we got the result of ultrasonography, five senior surgeons in our department decided the treatment strategies (operative and conservative treatment) referencing the pelvic stability for every patient.

When the final treatment plan was determined, patients were divided into an operative group and a conservative treatment group. In those patients who required surgery, either the ilioinguinal approach or the stoppa approach was selected. The operation was performed between days 3 and 10 post-injury. The plates (Matta pelvic system, Stryker) and the cannulated screws (6 mm-diameter ASNIS; Stryker, range from 55 to 85 mm) were used in the operation. As the conservative patients, following 3–4 days of pain relief, the patients were either sent home or to a community clinic, until the patient was tolerant of mobilization or weight-bearing on the affected side.

The follow-up frequency for these patients was at least once a month. All patients’ functions were evaluated using the Majeed grading system. In addition, X-ray images were used to observe healing or displacement. The time to weight-bearing was determined according to the degree of healing achieved.

### Statistical analysis

Statistical analysis was performed using SPSS (Version 25.0, SPSS Inc., Chicago, IL, USA). First, we assessed whether the measurement data was normally distributed using the Shapiro–Wilk test. We then analyzed the data using independent sample t-tests or the Mann-Whitney U test. The enumeration data was processed using the chi-square test. Differences were considered to be statistically significant if a *P* < 0.05 was obtained.

## Results

### Patient characteristics

In total, 77 patients with LC-1 pelvic fractures were included in the study. The average age of patients was 53.34 ± 15.91 years (range: 23–81 years). Electrocardiography monitoring was performed when the patients were admitted to the hospital, and we ensured that blood pressure and heart rates were stable.

There were 42 and 35 patients in TES and CUE group, respectively. The mean age was 52.26 ± 16.43 in TES group, 54.63 ± 15.40 in CUE group. The female took 52 % (22/42) and 43 % (15/35) in TES and CUE groups. The most of injury mechanism was accident in each group. The preoperative Visual analogue scale (VAS) was 3.05 ± 1.36 in TES group and 3.34 ± 1.49 in CUE group. There were no statistically significant differences in age, gender, injury mechanism, preoperative VAS, fracture types, or comorbidities between TES and CUE groups, showing Table 1.

**Table 1:** The baseline characteristics in TES and CUE groups

	TES group (n = 42)	CUE group (n = 35)	t/chi-square	*P*
**Age (years)**	52.26 ± 16.43	54.63 ± 15.40	-0.648	0.519
**Gender**				
Female	22	15	0.694	0.405
Male	20	20		
**Injury Mechanism**				
Accident	17	17	0.618	0.734
High falling	14	11		
Slipped	11	7		
**Preoperative VAS**	3.05 ± 1.36	3.34 ± 1.49	-0.907	0.367
**Fracture types**				
Unilateral pubic branch fracture	8	9	10.643	0.059
Bilateral pubic branch fracture	9	0		
Incomplete sacral + unilateral pubic branch fracture	8	10		
Incomplete sacral + bilateral pubic branch fracture	8	4		
Complete sacral + unilateral pubic branch fracture	6	7		
Complete sacral + bilateral pubic branch fracture	3	5		
**Comorbidities**				
Hypertension (%)	9	4	1.36	0.243
Diabetes (%)	3	5	0.42	0.517
Stroke (%)	1	3	0.494	0.482
Multiple injuries (%)	9	9	0.196	0.658

### Treatment for patients included in the study

In total, 44 patients received operation, including 29 (69 %, 29/42) in TES group and 15 (43 %, 15/35) in CUE group. There were less patients receiving the operative treatment in CUE group with statistically significant difference (chi-square = 5.347, *P* = 0.021). There were 14, 8, 7 patients used plate, screw, and plate plus screw in TES group, 12, 3, 0 patients in CUE group, respectively. In additional, there were 21 and 12 patients used stoppa and ilioinguinal approach, and minimally invasive treatment was used in 8 and 3 patients in TES and CUE group. As for the fracture types, the results shown that the distribution was different between two operative groups (chi-square = 13.093, *P* = 0.001). In the subgroup analysis, there was more patients with complete sacral plus unilateral/bilateral pubic branch fracture in CUE operative group than TES operative group, compared to the type of unilateral/bilateral pubic branch fracture (chi-square = 9.266, *P* = 0.002). There were no statistically significant differences in operative strategy and operative approach. As for the operative indicators, there were no statistically significant differences in operative time, blood transfusion, intraoperative liquid, and postoperative drainage in two groups. The average operative time was 114.43 minutes (range: 50–210). Even though there was no statistically significant difference in blood transfusion, the mean volume of blood transfusion in CUE group (1.33U) was more than that in TES group (0.90U). When to intraoperative blood loss, we found that the volume in CUE group (363.33 ± 190.74ml, range: 100-800ml) was more than that in TES group (268.28 ± 103.24ml, range:150-500ml) (t=-2.155, *P* = 0.037). Also, there was no differences in length of stay in hospital. However, the follow-up time in TES operative group was longer than CUE operative group (t = 12.047, *P* < 0.001). Furthermore, there were no differences in weight-bearing time, clinical healing time, or complications in two groups. When compared the Majeed scores in the operation population, we found that the mean score was 82.90 in TES group, and 83.73 in CUE group, and there was no statistically significant difference in the two groups, showing in Table 2.

**Table 2:** The operation subgroup in TES and CUE groups

	TES operation subgroup (*n* = 29)	CUE operation subgroup (*n* = 15)	t/chi-square	*P*
**Operative Strategy**				
Plate	14	12	5.532	0.063
Screw	8	3		
Plate plus Screw	7	0		
**Operative approach**				
Stoppa	19	9	1.769	0.413
IL	2	3		
Minimally invasive	8	3		
**Fracture types**				
Unilateral or bilateral pubic branch fracture	11	0	13.093	0.001
Incomplete sacral + unilateral or bilateral pubic branch fracture	13	5		
Complete sacral + unilateral or bilateral pubic branch fracture	5	10		
**Operative time (mins)**	112.41 ± 40.30	118.33 ± 58.27	-0.352	0.728
**Intraoperative blood loss (ml)**	268.28 ± 103.24	363.33 ± 190.74	-2.155	0.037
**Blood transfusion (U)**	0.90 ± 1.26	1.33 ± 1.45	-1.034	0.307
**Intraoperative liquid (ml)**	2072.41 ± 695.85	2146.67 ± 851.78	-0.311	0.758
**Postoperative drainage (ml)**	85.17 ± 64.12	115.33 ± 82.02	-1.343	0.186
**Length of stay in hospital (days)**	7.45 ± 1.88	7.73 ± 1.75	-0.487	0.629
**Follow-up time (months)**	23.03 ± 1.30	17.47 ± 1.73	12.047	< 0.001
**Weight-bearing time (months)**	1.03 ± 0.63	0.93 ± 0.26	0.755	0.455
**Clinical healing time (months)**	4.52 ± 1.81	4.13 ± 0.92	0.936	0.355
**Complications**				
Deep vein thrombosis	15	9	0.273	0.601
Superficial infection	2	1	0.000	1.000
Deep infection	1	1	-	1.000
Mortality	0	0	-	-
Majeed scores	82.90 ± 4.90	83.73 ± 12.46	-0.250	0.806

When compared the total population in two groups, we found that there were no statistically significant differences in length of stay in hospital, weight-bearing time, clinical healing time, complications (deep vein thrombosis), mortality between the two groups. As for the follow-up time, it was longer in TES group was than CUE operative group (t = 16.022, *P* < 0.001). More importantly, we found that the Majeed scores in CUE group (86.77 ± 9.72) was slightly higher than TES group (82.93 ± 4.90), with statistically significant difference (t=-2.125, *P* = 0.039), the Majeed scores in CUE conservative group (89.05 ± 6.48) was obviously higher than TES conservative group (83.00 ± 5.10), with statistically significant difference (t=-2.839, *P* = 0.008), showing Table 3.

**Table 3:** The outcomes and function in TES and CUE groups

	TES group (*n* = 42)	CUE group (*n* = 35)	t/chi-square	*P*
**Length of stay in hospital (days)**	6.50 ± 2.17	5.66 ± 2.24	1.675	0.098
**Follow-up time (months)**	22.98 ± 1.46	17.17 ± 1.72	16.022	< 0.001
**Weight-bearing time (months)**	0.71 ± 0.71	0.90 ± 0.42	-1.428	0.158
**Clinical healing time (months)**	4.45 ± 1.67	4.34 ± 1.14	0.341	0.734
**Complications**				
Deep vein thrombosis (%)	20	20	0.694	0.405
Mortality	0	0		
**Majeed scores**	82.93 ± 4.90	86.77 ± 9.72	-2.125	0.039
Conservative treatment	83.00 ± 5.10 (*n* = 13)	89.05 ± 6.48 (*n* = 20)	-2.839	0.008
Operative treatment	82.90 ± 4.90 (*n* = 29)	83.73 ± 12.46 (*n* = 15)	-0.250	0.806

**Fig. 2:**
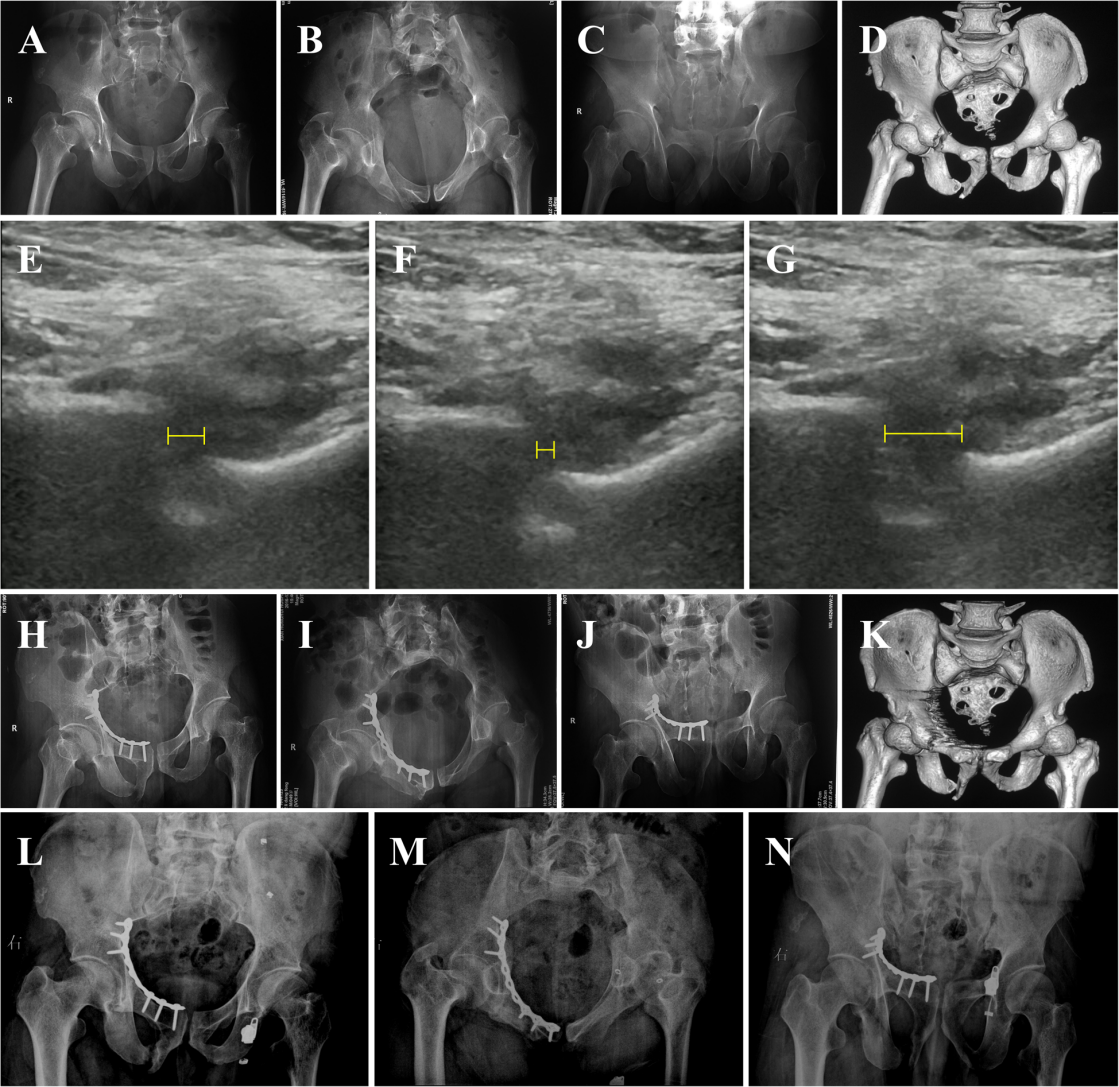
Case 1, 49-y-old male, fell from 3 m height. **a**-**c** Preoperative anterior-posterior, inlet and outlet X-rays; **d** Preoperative CT; **e** Displacement under ultrasonography when at rest; **f** Displacement under ultrasonography when compression; **g** Displacement under ultrasonography when separation. The left-right mobility was 0.72 cm and operation was chosen for the patient. **h**-**j** Postoperative anterior-posterior, inlet and outlet X-rays; **k** Postoperative CT; **l**-**n** Postoperative 11 months anterior-posterior, inlet and outlet X-rays. The patient recovered well and Majeed score was 90.

**Fig. 3:**
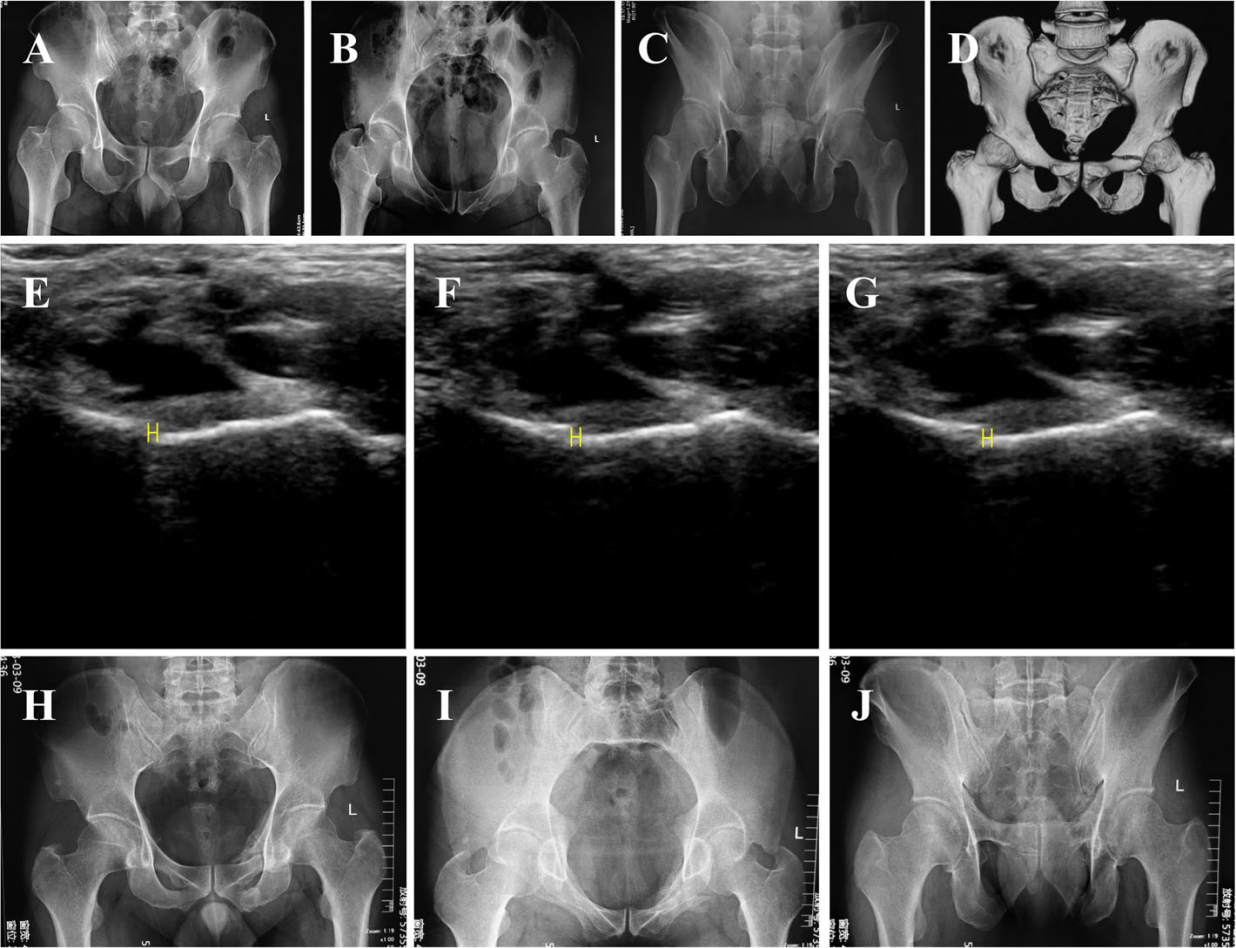
Case 2, 37-y-old male, suffered an accident. **a**-**c** Post-injury anterior-posterior, inlet and outlet X-rays; **d** Post-injury CT; **e** Displacement under ultrasonography when at rest; **f** Displacement under ultrasonography when compression; **g** Displacement under ultrasonography when separation. The left-right mobility was 0.03 cm and conservative treatment was chosen for the patient. **h**-**j** Post-injury 10 months anterior-posterior, inlet and outlet X-rays. The patient recovered well and Majeed score was 94.

There were two case presentations in Fig. 2 and Fig. 3.

## Discussion

The stability of pelvis is vital important to the sequential treatment in LC-1 fracture, but determining the stability is very difficult and various in surgeons. In our previous studies [9, 10], we only found that left-right mobility ≥ 0.3 cm may be used as the criterion for determining instability under the ultrasonography examination. There is no evidence that CUE strategy could identify the real unstable and stable pelvis, and the overall function of LC-1 patients who used CUE strategy are better than TES strategy. Therefore, this historical control study is designed to verify this question. Our basic findings were following: **(a)** There were less proportion patients in CUE group chosen operative treatment after the ultrasonography examination. **(b)** The intraoperative blood loss in CUE operative group was more than TES operative group. **(c)** There were more complete sacral fractures in CUE operative group than TES operative group. **(d)** The Majeed scores in CUE conservative group was higher than TES conservative group. **(e)** The overall Majeed scores in CUE group was higher than that in TES group.

As the treatment strategy, the proportion of receiving operation in CUE group was (43 %) less than TES group (69 %). In the study of Sagi et al., they found 65 % LC-1 fractures were stable and 35 % were unstable based on the instability during the examination with fluoroscopy under anesthesia [8]. On the basis of there was no significant difference in the distribution of fracture types, and the proportion of needing surgical patients in the CUE group was lower than TES group. We thought the possible reason is that the most of LC-1 pelvic rings are really stable and it is conservative treatment that can be adopted. In other words, ultrasonography examination may identify the real unstable LC-1 pelvis. Thus, the operation rate in CUE group is reduced.

As for the operative strategy, the minimally invasive treatment was considered firstly and following by open reduction and internal fixation. So far, transiliosacral screw was recognized as one of the standard procedures for the treatment of unstable posterior pelvic ring lesions [12, 13]. Eight patients in TES group and 3 patients in CUE received minimally invasive treatment only. Even though there was no statistically significant difference in blood transfusion, there was a tread that the mean volume of blood transfusion in CUE group (1.33U) was more than that in TES group (0.90U). Significant blood loss from pelvic fracture is possible because of the rich arterial and venous channels, the plentiful blood supply of the pelvic bones, and the fact that tissue pressure within the pelvic retroperitoneum is low [2]. Usually, patients with different degree of stability of pelvic fractures had various transfusion volume, the more instability, the more volume of transfusion [14]. In addition, the volume of intraoperative blood loss in CUE operative group was more than that in TES operative group. The volume of blood transfusion and intraoperative blood loss maybe indicate the stability of the pelvic ring. Thus, in this controlled study, the real unstable pelvis from CUE group may have more volume of blood transfusion and intraoperative blood loss than the mixed stable and unstable pelvis in TES group.

As for the fracture types, the result showed that the distribution was different between two operative groups. When a subgroup analysis was introduced, we found there was more complete sacral fractures in CUE operative group than TES operative group. This phenomenon is corresponded to the previous studies: LC-1 injuries with a complete posterior sacral injury could definitely gain benefit from surgical stabilization [15], and those with a complete sacral fracture and bilateral rami fractures displace at a significantly greater rate [16]. This fact reflects that CUE strategy could relatively accurately identify the unstable LC-1 pelvis than TES strategy.

When to the field of function, it is a vital indicator in this controlled study. The results showed the function in CUE conservative group was better than TES conservative group, and the overall function in CUE group was also better than that in TES group. The possible reason is that there are mixed stable and unstable pelvis (needing operation) in TES conservative group, but all of these patients are given the non-operative treatment. On the contrary, the conservative patients in CUE group are mainly stable pelvis and given the suitable treatment. Thus, Majeed scores in CUE conservative group was higher than TES conservative group. Further, the patients in CUE group are received the relative suitable treatment strategy, so the overall Majeed score is higher. However, this superiority is only found in a trend between two operative groups, without statistically significant difference, the Majeed scores in CUE operative group (83.73 ± 12.46) was similar to the TES group (82.90 ± 4.90). The one possible reason is the operative sample in this study is 44, another possible reason is that the follow-up in CUE group is shorter than the TES group.

The follow-up time was different in two groups, which was determined by the methodology of the study. The follow-up time of the CUE group was 6 months shorter than that of the TES group, but the overall function in CUE group was better than TES group, which could show the advantages from ultrasonography and explain the necessity of ultrasonography examination to distinguish the stability of the pelvic ring. To further explore this issue, it is necessary to observe the functional results of the two groups in a longer follow-up period.

Even though, there is a proposed scoring system was in predicting nonoperative or operative treatment, based on radiographic fracture characteristics of the sacrum, inferior ramus, and superior ramus [17]. Individual characteristic on the initial static X-ray and CT scan images of LC-1 pelvis vary greatly, and it is difficult to distinguish stability in imaging data. Therefore, it is difficult to know which patients are suitable for surgery and which patients are suitable for conservative treatment. The ultrasonography examination is a useful method and could identify the real unstable and stable pelvis, and the overall function of LC-1 patients could be improved under ultrasonography examination.

It is worth mentioning the limitations in this study. This study is designed as historical control study in different period. Even though the operation is carried out by one team, the operative experience may increase in the CUE group.

## Conclusions

In conclusion, the ultrasonography examination could relatively accurately identify the unstable LC-1 pelvis than the traditional experience strategy, the operative rate could be reduced and the overall function of LC-1 patients could be improved under the ultrasonography examination.

## Data Availability

The datasets generated during and/or analyzed during the current study are not publicly available due to data privacy but are available from the corresponding author on reasonable request.

## Abbreviations

LC: Lateral compression
CT: Computed tomography
VAS: Visual analogue scale
